# Lifespan-Associated Gene Expression Signatures of Recombinant BXD Mice Implicates *Coro7* and *Set* in Longevity

**DOI:** 10.3389/fgene.2021.694033

**Published:** 2021-07-09

**Authors:** David Vitiello, Alexander Dakhovnik, Cyril Statzer, Collin Y. Ewald

**Affiliations:** Laboratory of Extracellular Matrix Regeneration, Department of Health Sciences and Technology, Institute of Translational Medicine, ETH Zürich, Schwerzenbach, Switzerland

**Keywords:** healthy aging, BXD mice, gender-specific lifespan, coronin, SET, longevity, omics

## Abstract

Although genetic approaches have identified key genes and pathways that promote longevity, systems-level approaches are less utilized. Here, we took advantage of the wealth of omics data characterizing the BXD family of mice. We associated transcript and peptide levels across five tissues from both female and male BXD isogenic lines with their median lifespan. We identified over 5000 genes that showed a longevity correlation in a given tissue. Surprisingly, we found less than 1% overlap among longevity-correlating genes across tissues and sex. These 1% shared genes consist of 51 genes, of which 13 have been shown to alter lifespan. Only two genes -*Coro7* and *Set*- showed a longevity correlation in all tissues and in both sexes. While differential regulation of aging across tissues and sex has been reported, our systems-level analysis reveals two unique genes that may promote healthy aging in unique sex- and tissue-agnostic manner.

## Introduction

In the last 30 years, genetic approaches have identified key genes and pathways that promote lifespan extension across species (Kenyon, 2010; Singh et al., 2019; Zhang et al., 2020). Valuable molecular insights came from loss- and gain-of-function genetic alterations in model organisms that usually have a genetically uniform background. In general, genetically uniform C57BL/6 inbred male mice are used for aging research, and about 90% of biomedical research has been performed on male C57BL/6 and sub-strain mice (Walsh et al., 2014; Selman and Swindell, 2018). The advantages of using the same wild-type mouse strain with a uniform genetic background for biomedical research make findings comparable and reproducible. However, humans are far from a uniform genetic background. Translating findings from inbred mice need to be confirmed in outbred lines or intercrossed inbred lines. Inbred mice strains might be confounded by altered genetic and metabolic pathways from genetic drift, inbreeding depression, growth adaption, and loss of genetic and metabolic robustness (Selman and Swindell, 2018). For instance, dietary restriction, which increases lifespan across many model organisms and even exotic species (Mair and Dillin, 2008; Fontana et al., 2010), is less effective in the inbred wild-type DBA/2 mouse strain compared to the about 30% lifespan increase of C57BL/6 mice (Turturro et al., 1999; Forster et al., 2003; Swindell, 2012; Mitchell et al., 2016). Under *ad libitum* fed conditions, DBA/2 shows about 25% shorter mean lifespan compared to C57BL/6 mice (Mitchell et al., 2016). Interestingly, the amount of caloric restriction (CR) also plays a role with 20% and 40% CR having similar lifespan extension effects in DBA/2 mice, while 20% CR is far superior in C57BL/6 mice (Mitchell et al., 2016). The difference in genetic background and response to CR of these two inbred wild-type mouse strains offers an excellent opportunity to dissect the molecular makeup underlying their differences in aging and lifespan.

Starting in 1973, the C57BL/6J were crossed with DBA/2J mice to generate more than 140 fully isogenic BXD mouse strains (Ashbrook et al., 2021). These BXD lines have different genetic backgrounds mirroring the complexity of human populations (Peirce et al., 2004; Ashbrook et al., 2021). BXD strains are one of the best-characterized models, with their genome being fully sequenced, quantification of transcriptomics, proteomics, and metabolomics of several tissues, and over seven thousand phenotypes recorded (Ashbrook et al., 2021). Although the difference in the mean lifespan of the parental strain (C57BL/6 and DBA/2) is only about 25% (Mitchell et al., 2016), these BXD inbred strains show more than twofold differences in lifespan (Roy et al., 2020). This makes them unique in order to identify longevity genes based on quantitative trait loci (QTL) mapping (Gelman et al., 1988; Haan and Zant, 1999; Lang et al., 2010; Houtkooper et al., 2013).

Here, we took advantage of the wealth of omics data of these well-characterized BXD strains. We hypothesized that by correlating tissue- and sex-stratified gene expression signatures with lifespan, we could identify novel longevity-promoting genes. Our analysis reveals several novel and known-longevity-promoting genes. We find little overlap of lifespan-associated gene expression across tissues and observe sex-specific differences within the same tissue. Only two genes (*Coro7 and Set*) show lifespan-correlating gene expressions across tissues.

## Materials and Methods

### Data Collection

The data sources employed are available on GEO and gene network 2 (gn2.genenetwork.org) and linked in Supplementary Tables 1, 2. All the collected data involves experiments within the BXD family and mm10 in the search tool (Mulligan et al., 2017; Watson and Ashbrook, 2020). Three datasets included male mice experiments, one both sexes, and three came from experiments on female mice. The data was already normalized with RMA or RSN, as indicated in gn2.genenetwork.org or in Supplementary Table 1.

#### Adrenal Gland

Young adult mice raised in the specific pathogen-free vivarium at UTHSC. Whole adrenal glands were collected from young adult mice. In general, sex-balanced samples (one array from male and one array from female) for BXD and parental strains were performed. The GeneChip^®^ Mouse Gene 1.0 ST Array was used to analyze the data. More information at http://gn1.genenetwork.org, gn accession ids: (female) 426, (male) 425.

#### Kidney

The data was extracted from the July 2006 Kidney QTL Consortium data set. It provides normalized mRNA expression levels in the adult kidney of 70 genetically diverse strains of mice including 54 BXD recombinant inbred strains. Kidney samples were processed using a total of 153 Affymetrix Mouse Expression 430 2.0 microarrays (M430v2.0). BXD animals were obtained from UTHSC, and the Jackson Laboratory (Supplementary Table 1). Mice were housed at UTHSC, at Harvard/BIDMC, at the University of Memphis, or the Jackson Laboratory before sacrifice. Kidneys were dissected whole and cleaned from the adrenal glands. Kidneys from two to six animals per strain were pooled.

BXD mice used in this study were between 50 and 99 days of age (accessed July 2020)^1^. The data set consists of arrays processed in twenty-three groups over a 6 months period (March 2006 to July 2006). Each group consists of 4 to 12 arrays. All arrays were processed using the Affymetrix Eukaryotic Sample and Array Processing protocol (701024 Rev. 3). The Affymetrix Mouse Genome 430 2.0 array was used to generate the data. Further details are available at http://gn1.genenetwork.org/webqtl/main.py?FormID=sharinginfo&GN_AccessionId=239&InfoPageName=MA_M2F_0706_R (accessed July 2020).

#### Bone Femur

RNA from cortical bone (femoral diaphysis free of marrow) were profiled from 99 Hybrid Mouse Diversity Panel strains to be profiled. Sixteen-week-old male mice were used in this study. A total of 1–3 mice per strain were arrayed. The dataset is part of the bone microarray profiles from the Hybrid Mouse Diversity Panel.

#### Eye

Data were extracted from the HEIMED September 2008 RMA data release, which provides normalized gene expression levels of whole eyes of 103 BXD lines of young adult mice generated using 221 Affymetrix M430 2.0 arrays. On average, collectively six eyes of the same BXD strain with common sex and age were pooled for the microarray. Data were generated at UTHSC using pooled RNA samples, usually two independent pools for males and females, for most lines of mice. This data set was processed using the RMA protocol. A total of 2223 probe sets are associated with LRS values greater than 46 (LOD > 10) (accessed July 2020)^2^. Further details about the protocol are available at http://gn1.genenetwork.org/webqtl/main.py?FormID=sharinginfo&GN_AccessionId=207&InfoPageName=Eye_M2_0908_R (accessed July 2020).

#### Liver

The Data consists of 89 BXD strains. Mice were maintained in the UTHSC vivarium in Specific Pathogen-Free (SPF) housing. Diet used for this analysis was *ad libitum* fed Harlan Teklad 2018 (CD; 18.6% protein, 6.2% fat, and 75.2% carbohydrates). BXD mice were sacrificed at specific ages for liver collection. The majority of mice lived out their normal lifespan. For details, [see Williams et al. (2021)].

### Data Analysis

Transcript or protein levels were normally represented as z-scores, with the exception of sets coming from Gene Network 2, some of which had employed a different convention, named thereby 2z + 8 score. This corresponds to a rescaling of the data to a mean of 8 units with a standard deviation of 2 units. The average sample size across all datasets was 16, while the age at which tissues were collected was on average 25 weeks (average age within the different sets ranging from 8 to 60 weeks). We used R Cran 3.6.0. to conduct the *in silico* analysis. The matched strains were then ordered by shortest to longest-lived, and for each set, the four shortest- and four longest-lived strains were selected for further downstream analysis, on the assumption that these would show the most dramatic changes in those genes which are important determinants of lifespan.

To define the longevity genes landscape across all given tissues, we assessed the correlation of transcriptomics and proteomics records from seven different datasets from five different mice tissues, adrenal gland, bone, kidney, eye, and liver, with median lifespan (referred herein as “correlation with longevity”). For all of the seven datasets, we first matched each BXD mouse strain with the lifespan values from a reference QTL longevity analysis (Lang et al., 2010). We compared the Lang *et al.*, 2010 BXD lifespans ranking (Lang et al., 2010) with a prior QTL study by Gelman *et al.*, 1988 (Gelman et al., 1988), which provided median lifespans on BXD mice, assessing the median lifespans of 20 RI BXD strains from 360 female mice (∼ 20 mice for 16 strains and 10 mice for 4 strains). The authors came to similar results for female median strains, with a correlation between the relative median lifespans of 0.73 at a *P* < 0.001 (Lang et al., 2010). The average sample size across the datasets employed in our analysis amounts to 43 (Supplementary Table 1), which corresponds to a critical *r*- value of 0.349 at a *P*-value of 0.05.

**FIGURE 1:**
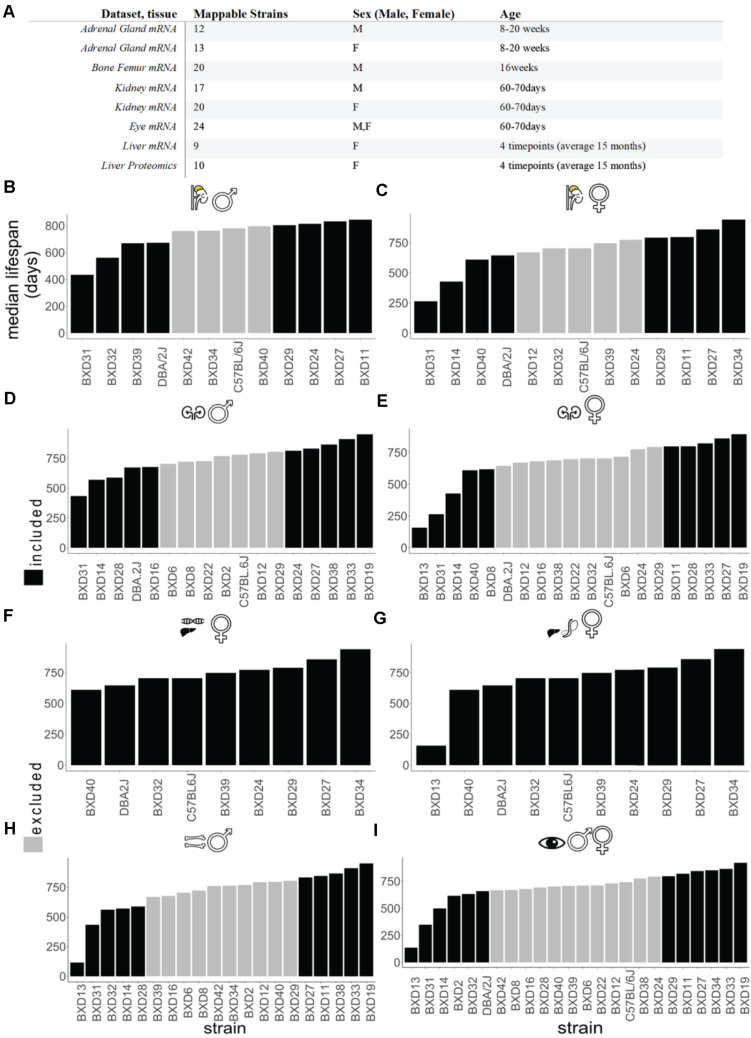
Data landscape of BXD mice strains and lifespan. **(A)** The selection of datasets for the *in silico* analysis. All of the sets listed are downloaded from gene network 2 or GEO databases (http://gn2.genenetwork.org/ either directly or by the reference GEO accession). The strain’s total amount refers to the subset of the study strains which could be mapped to median strain lifespan values. See Supplementary Tables 1, 2 for details. **(B–I)** Ordered strain median lifespans for each tissue. Adrenal gland male **(B)** female **(C)**, kidney male **(D)**, female **(E)**, liver female for transcriptomics **(F)**, liver female for proteomics **(G)**, bone femur male **(H)**, and sex-combined eye tissue **(I)**. The analyses encompass the subset of BXD strains which, within each dataset, were mappable to the employed experiment for BXD lifespans. In black are the top four shortest and longest-lived BXD mice strains.

The list of strains employed for each dataset and that were employed in the correlation analysis in each case are listed in Figure 1A. We thus opted for a *Pearson’s r* lower bound of 0.4, as it represents a reasonable choice in accordance with the aforementioned critical r value determined using Student’s t distribution. This value has been used throughout the analysis to make a distinction between better and worse longevity correlated genes, with 0.7 used sometimes to restrict the analysis to the top longevity correlated genes. Next, we proceeded to compute Pearson’s correlation coefficient (r) values to correlate the strains’ median lifespan with gene expression/peptide abundance.

For correlations, we decided to employ Pearson’s rather than Spearman’s correlation, since the latter ranking step of lifespan values results in the flattening out of the differences amongst the strains. In fact, since lifespan values in this context belong to a continuous and non-ordinal scale, a parametric test represents a safer approach in estimating correlation. Because the average sample size across the experiments is small, employing a non-parametric test as Spearman is unlikely to identify non-monotonic relations between variables. The correlation values range from 0 to + ⁣− 1. In accordance with the widely accepted categorization of effect sizes by Cohen (1977) and a more recently loosened-up grouping by Gignac and Szodorai (2016), we decided on an absolute *r* of + 0.4 as being a satisfying longevity score (in accordange to the previously tested r-critical value). The scored genes were then vetted, and -omics items scoring absolute *r* values below + 0.4 were excluded.

**FIGURE 2:**
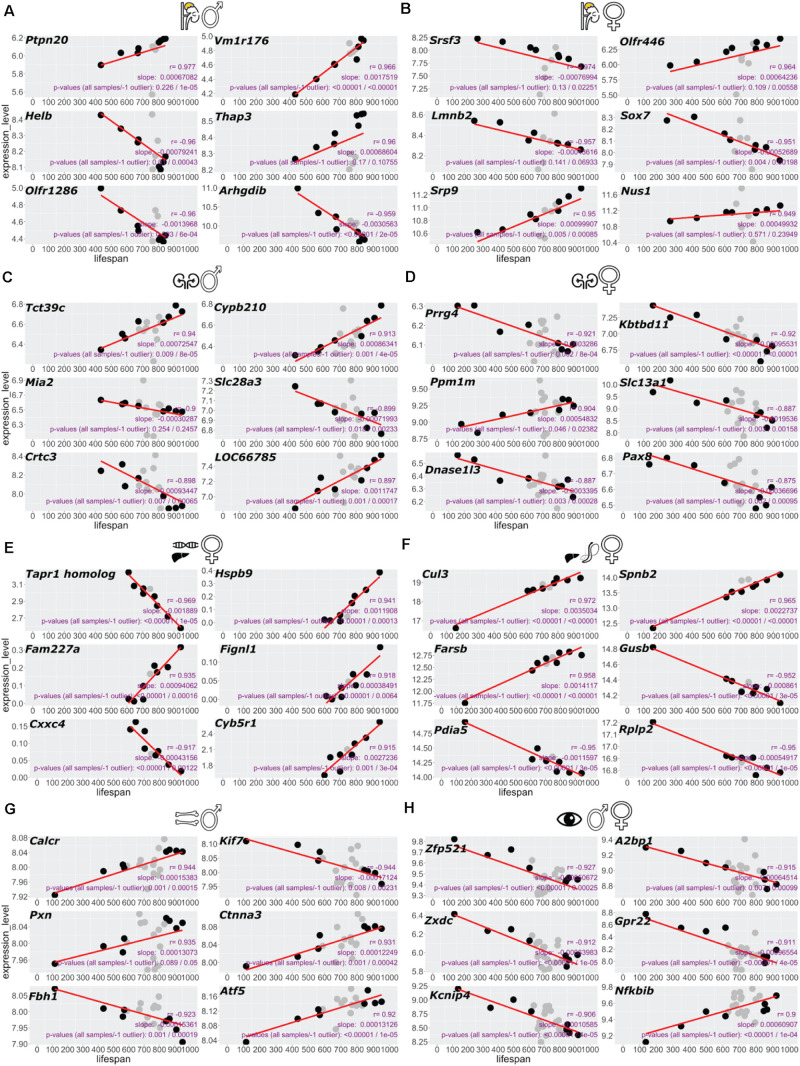
Top six genes correlating with BXD lifespan in each dataset. Gene expression correlating with lifespan scored by highest Pearson’s R for each tissue. Adrenal gland male **(A)**, female **(B)**, kidney male **(C)**, female **(D)**, female liver RNA-seq **(E)**, liver female proteomics **(F)**, bone femur male **(G)**, and sex-combined eye tissue **(H)**. **(A–E,G,H)** RNA-seq or microarray data and **(F)** proteomics data.

We regressed lifespan against expression for each gene or protein of interest using a univariate linear model and reported the slopes of the fitted models in Figures 1, 2, 6. To identify overarching longevity top hits, we merged the genes-lifespan correlation values from the various sets and ranked them according to both their presence in tissues and average correlation value, *r*. We used biomaRt v. 2.4.5 (Durinck et al., 2009) with the Ensembl archive release 100 for converting between gene annotations.

We used ClusterProfiler v. 3.90 (Yu et al., 2012) to generate GO enrichments of the tissue with these longevity-correlating genes. Background mmu genome was used whenever possible; no background enrichment was otherwise used. The percentage estimation of the overlapping longevity hits across tissues was carried out with the eulerr 6.1.0 (Larsson, 2020) R package.

To determine the mRNA expression levels of *Coro7* and *Set* upon longevity interventions, we analyzed publicly available expression datasets to quantify the change of *Coro7* or *Set* expression levels in long-lived compared to normal-lived mice as described in (Statzer et al., 2020).

## Results

### Omics Data and Parsing BXD Stains Based on Their Median Lifespan

To identify gene expression levels correlating with lifespan, we took advantage of the well-characterized BXD mouse family and their publicly available omics data^3^. Our analysis included proteomics data from female livers and transcriptomic data of various tissues (adrenal gland, bone, kidney, eye, and liver) from both males and females fed on a standard CHOW diet (Figure 1A and Supplementary Table 1). To stratify this data according to how long an isogenic BXD inbred line has lived on average, we ranked their median lifespan from shortest to longest-lived strains (Figures 1B–I and Supplementary Table 1) (Lang et al., 2010). We observed different orders of median lifespan ranking of BXD strains for both sexes (Figures 1B–I), which is consistent with sex differences reported in most inbred strains.

### Gene Expression Correlating With Lifespan

To establish a “longevity” parsed comparison, we reasoned that the strains on the far ends of the median ranked lifespan would hold the most valuable information. Given that not all BXD strains were analyzed for transcriptomics or proteomics of a given tissue (Figure 1A), we defined the four shortest and longest-lived BXD strains as the longevity comparison dataset used here, except for the liver, where we used the entire dataset due to a lower number of strains profiled in this tissue (Figures 1B–I and Supplementary Table 1). Using this approach, we calculated the slope of the linear regression (s) and Pearson’s correlation (*r*) for each transcript or peptide (Supplementary Table 2). For each tissue, we ranked gene and protein expression levels by the highest correlation (Figure 2 and Supplementary Table 2). From the top six genes of each tissue, we did not find any shared genes but detected *Srsf3, Lmnb2, Crtc3, Slc13a1/Nas1, Pax8, Atf-5, Zfp521*, and *A2bp1* known to be implicated in longevity (Figure 2 and Supplementary Table 2).

**FIGURE 3:**
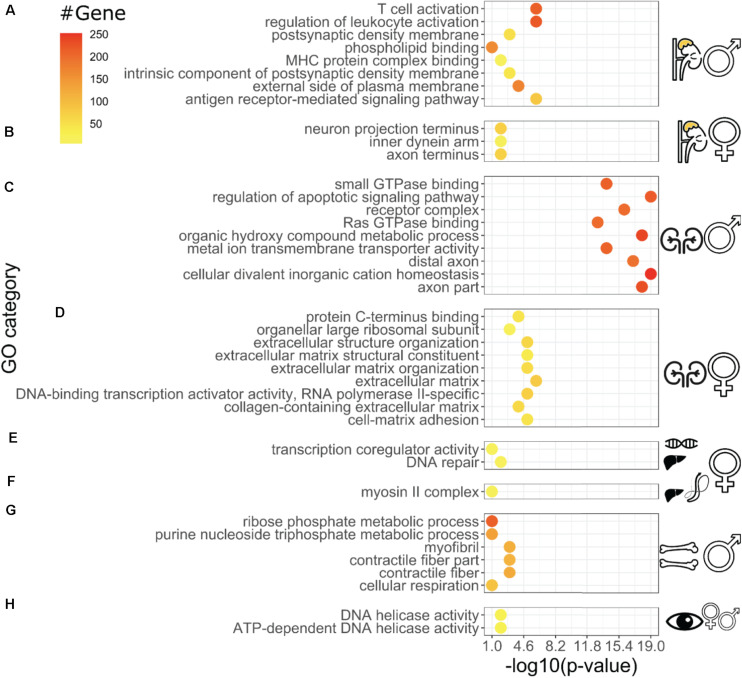
Distinct Gene Ontology enrichments of longevity-correlating genes for individual tissues Gene Ontology categories (Biological Process, Molecular Function, and Cellular Component) with *P*-values for each tissue. Adrenal gland male **(A)**, female **(B)**, kidney male **(C)**, female **(D)**, female liver RNA-seq **(E)**, female liver proteomics **(F)**, bone femur male **(G)**, and sex-combined eye tissue **(H)**. For each dataset, the genes that were most correlated with longevity have been analyzed for clusters of enrichments in the Gene Ontology categories. The display of difference within the same tissues **(C,D)** and -omics type **(E,F)** illustrates how longevity might be dysmorphic based on sex and biochemical processes involve (i.e., longevity secretome vs. longevity transcriptome) even within the same experiment **(E,F)**. The *P-*values on the *X*-axis measure the significance of the enrichment categories shown on the *Y*-axis, and are computed based on, amongst others, the number of genes belonging to the same GO category (“count,” top-right legend), which are color-coded in the data points. More details regarding the enrichment procedure are given in the section “Materials and Methods.”

We observed tissue-specific gene ontology (GO) enrichment of the best longevity correlated genes (Figure 3 and Supplementary Table 3). These include several processes known to be vital to promote healthy aging, such as immune responses, neuronal signaling, heat shock proteins, epigenetic reprogramming, extracellular matrix, and mitochondrial energy metabolism (Figure 3; Greer et al., 2011; Houtkooper et al., 2013; López-Otín et al., 2013; Ewald et al., 2015; Denzel et al., 2019; Ewald, 2019; Hess et al., 2019; Vertti-Quintero et al., 2019; Gallotta et al., 2020). For instance, the adrenal gland showed enrichment for immune responses, whereas in the liver, oxidative phosphorylation and mitochondrial components were enriched (Figure 3). In addition, we observed sex-specific differences in gene ontology enrichment (Figure 3). For instance, in the male adrenal glands, longevity correlated with immune response, whereas in the female adrenal glands longevity correlated with neuronal innervation (Figures 3A,B). Similarly, Ras signaling was enriched for longevity in the male kidney, whereas extracellular matrix organization was enriched in the female kidney (Figures 3C,D). Taken together, these data suggest that each tissue modulates gene expression in a distinct way as a function of lifespan which may be sexually dimorphic.

### Less Than 1% Overlap of Individual Gene Expression Associated With Longevity

**FIGURE 4:**
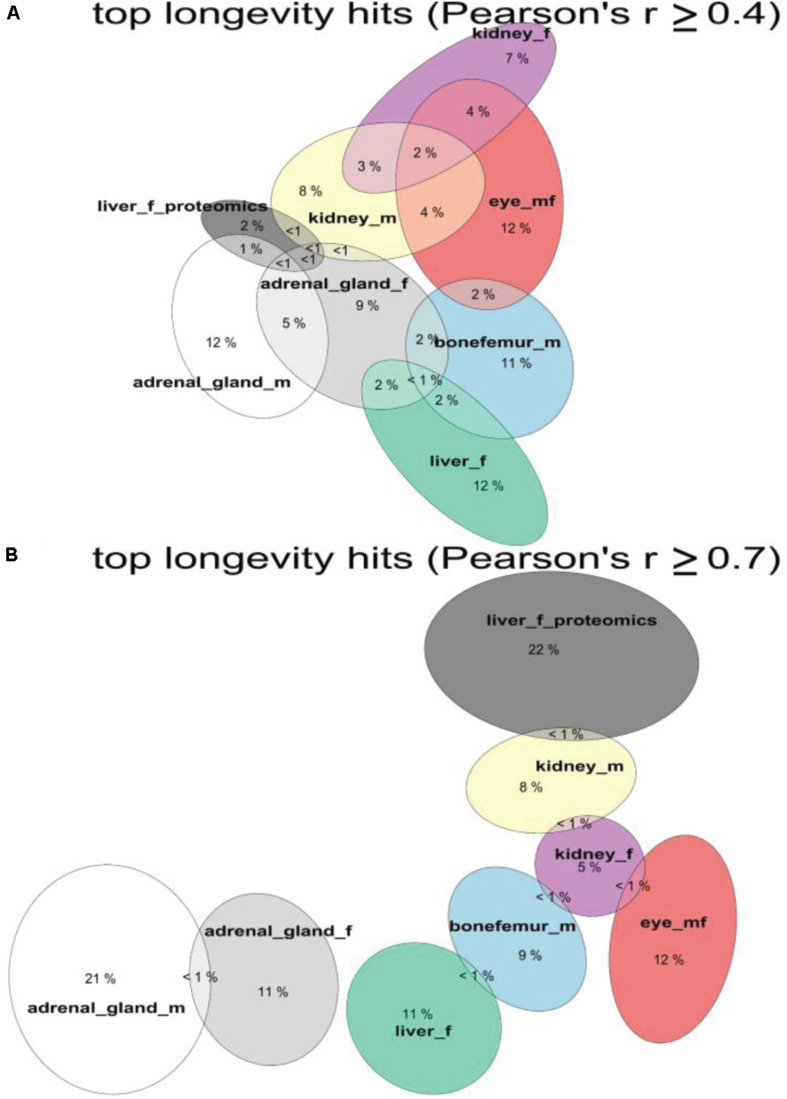
Shared longevity-correlated genes from different tissues. The overlap in percentages of longevity-correlating genes and proteins among tissues and datasets. Overlap with threshold of Person’s | r| ≥ ± 0.4 for **(A)** and | *r*| ≥ ± 0.7 for **(B)**. See Supplementary Table 4.

To determine whether there would be any longevity-associated genes that would be shared among tissues, we used two Pearson’s correlation cutoffs of *r* > 0.4 and *r* > 0.7 (Figure 4 and Supplementary Tables 1, 2, 4). With a Pearson’s correlation cutoff of *r* > 0.4, we found that liver proteomics had the most overlap across tissue transcriptomics levels (Figure 4A). About 15–30% of all total transcripts showed longevity correlation in individual tissues and sexes (Supplementary Table 4). However, less than one percent was shared among individual tissues. Furthermore, liver proteomics showed no overlap with liver transcriptomics (Figure 4A). Similarly, the liver proteomics and transcriptomics did not show any overlap when we used a Pearson’s correlation cutoff of *r* > 0.7 (Figure 4B), even though the data comes from the same experiment (Williams et al., 2021). Strikingly, there were no common signature genes among these tissues (Figure 4B). At the *r* > 0.7 cutoff, the overlap between two tissues or with the same tissue of opposite sex was less than one percent (Figure 4B), suggesting potential distinct sex- and tissue-specific processes are adopted to assure healthy aging among these isogenic inbred strains.

### Only a Few Shared Genes Across Tissues Correlated With Lifespan

We next wondered which of these less than 1% genes were shared among tissues. To address this we used Pearson’s correlation cutoffs of *r* > ± 0.4 and identified 51 shared genes (Supplementary Table 5). Out of these 51 genes, 13 had previously been directly implicated in aging, or their orthologs in other species had shown altered lifespan (Supplementary Table 5). For instance, genetic alterations in orthologous genes such as *App, Ndufc2, Coro7, Trap1*, and *Mtf1* have previously been shown to increase the lifespan of *C. elegans* or *Drosophila* (Supplementary Table 5). In humans, mutations in *Banf1* result in progeria (Jamin and Wiebe, 2015), whereas single nucleotide polymorphisms in *Cnksr2* are associated with human longevity (Wright et al., 2019). Moreover, copy number variations in *Cntnap2* are also associated with healthy aging (Iakoubov et al., 2014) (Supplementary Table 5). These 51 genes were mainly enriched for neuronally expressed genes (*Cnksr2, Dlg2, Magi2, Exoc4, Dclk1*, and *App*) and mitochondrial genes (*Pold3, Trap1, Chchd3, Psmb3, Tomm70a, Ndufc2, Hars*, and *Gdap1*; Supplementary Table 5). Since our analyzed datasets did not include any neuronal tissues, we confirmed that these genes are expressed in non-neuronal tissues using the Mouse Gene Expression Database (Smith et al., 2018) (Supplementary Figure 1). This suggests that these neuronal genes might have a potential role in longevity in non-neuronal tissues, although further investigations into this are required.

### *Coro7* and Set Are Shared Among All Seven Tissues

**FIGURE 5:**
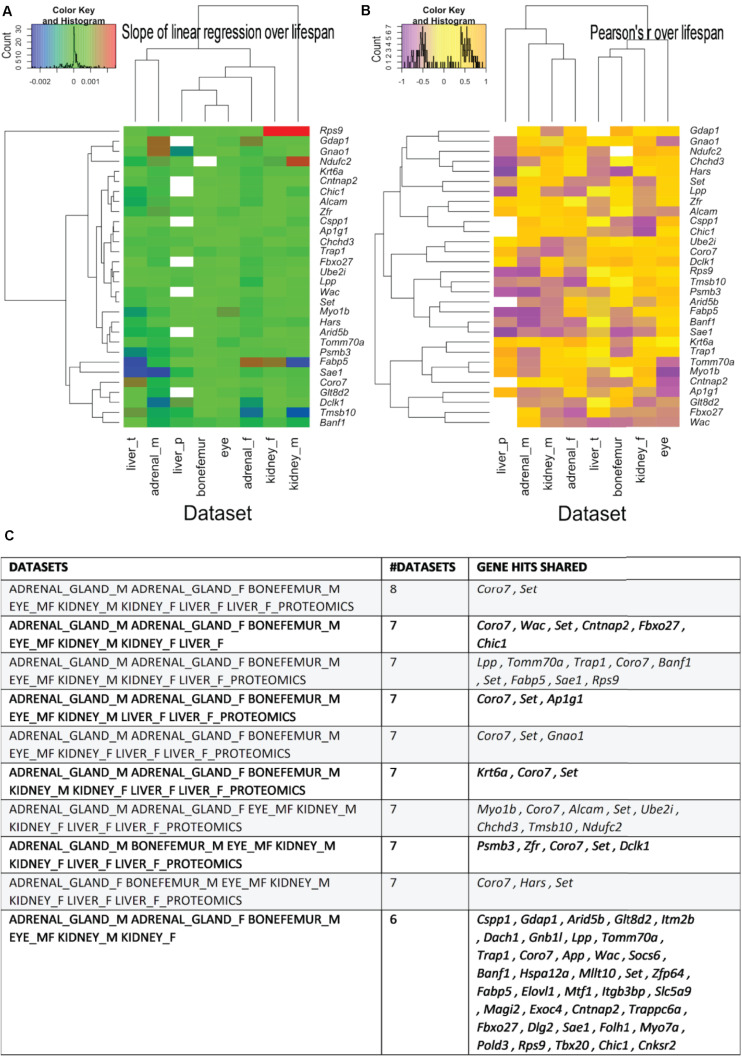
The most shared genes correlating with lifespan. **(A)** Heatmaps showing the clustered common longevity genes by both the values of the linear regression over median strain lifespans, respectively. **(A)** Pearson’s correlation coefficient over the median strain lifespans **(B)** and tissue of origin **(A,B)**. The commonality is here intended as being present in the source -omics data with a Pearson’s *r* absolute value greater than 0.4 and in more than one data set. Particularly, the genes used for generating the heatmaps are the ones that are present in most tissues and are shown in panel **C**, with the indication (left column) of the dataset they come from. See Supplementary Table 6 for a more detailed listing of the genes.

**FIGURE 6:**
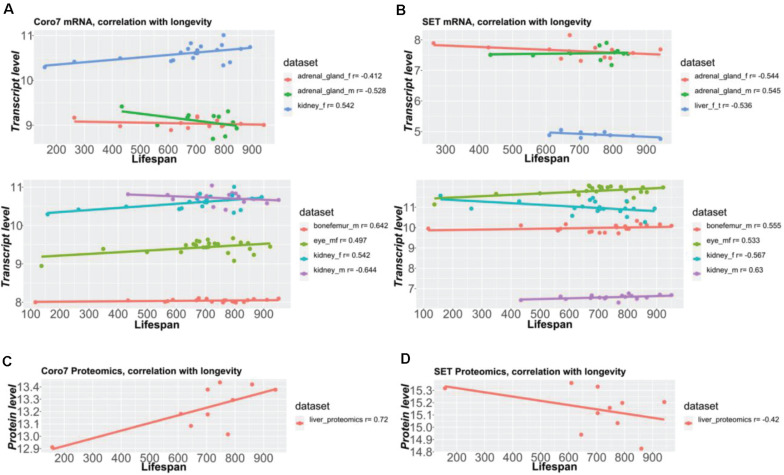
*Coro7* and *Set* gene expression correlate with lifespan across tissues. Coronin 7 **(A,C)** and SET nuclear oncogenes **(B,D)** were identified as the only two pervasive longevity genes, across all eight datasets with a longevity score (absolute Pearson’s *r*) ≥ ±4, SET being the best longevity-correlated gene (0.53 < | *r*| < 0.63 across tissues), followed by Coronin-7 (0.41 < | *r*| < 0.64 across tissues). The *Y*-axis values represent normalized values (**A,B**: bottom parts) via RMA (except bone femur, normalized via VST + RSN) or 2z + 8 scores (**A,B**, upper parts, **C,D**) (see Supplementary Table 1).

Next, we sought to determine the shared genes among tissues that showed expression profiles correlating with longevity. From the 51 shared genes, we identified the top ten shared genes across tissues (Figure 5A and Supplementary Table 6). We found that 38 and 26 genes were shared among six and seven datasets, respectively (Figure 5B and Supplementary Table 6). Among all eight datasets of the five tissues from both sexes, only two genes were shared, *Coro7* and *Set* (Figure 5). Coronin-7 (*Coro7*) is essential for cytoskeletal functioning (Park et al., 2021). *Set*, or SET nuclear oncogene, works by inhibiting acetylation of nucleosomes, especially histone H4, by histone acetylases (HAT) (Ashbrook et al., 2021). We observed both positive and negative *Coro7* and *Set* expression levels associated with longer-lived BXD strains in a tissue-specific manner (Figure 6). We next determined the mRNA levels of *Coro7* and *Set* in different wild-type mice upon genetic or pharmacological interventions that increase lifespan (Supplementary Figures 2, 3). We found that *Coro7* and *Set* were differentially expressed across longevity interventions. However, the directionality of expression appears to be sex- and tissue-specific (Supplementary Figures 2, 3 and Supplementary Table 7). This suggests that both genes respond to nutritional, pharmacological, and genetic longevity-promoting interventions.

## Discussion

Genetic inbred lines, such as the BXD mouse strains, are powerful tools to gain molecular insights into a trait of interest. Here we asked whether there are genes, which expression levels would correlate with the duration of the lifespan of individual BXD mouse strains. We observed unique tissue-specific and sex-specific gene expression signatures correlating with lifespan but only observed a few gene expression signatures shared among tissues. Importantly, there was an enrichment for longevity-promoting genes in either the top gene candidates of individual tissues or the gene hits shared across multiple tissues. We identified only two gene expression signatures - *Coro7* and *Set*- that correlated with longevity across all seven tissue datasets.

Although the nuclear oncogene Set controls many processes, such as DNA-damage repair and cancer, important for preventing age-related damages or pathologies (Kalousi et al., 2015; Bayarkhangai et al., 2018), no direct evidence of Set controlling lifespan exists. By contrast, inhibition of *Coro7* extends *C. elegans* lifespan (Kim and Sun, 2007). The coronin *Coro7* is well conserved from *Dictyostelium* to humans (Chan et al., 2011). *Dictyostelium* coronin mutants show defects in endocytosis/phagocytosis (Maniak et al., 1995). Similarly, *Coro7/pod-1* loss-of-function *C. elegans* mutants accumulate large endocytic vesicles and extracellular deposits during embryogenesis, suggesting defects in endocytosis and removal (Rappleye et al., 1999). Furthermore, *C. elegans Coro7/pod-1* binds actin to establish the anterior-posterior axis of embryos (Rothenberg et al., 2003). The *Drosophila Coro7/pod-1* (*dpod1*) crosslinks actin and microtubule for neuronal axon pathfinding (Rothenberg et al., 2003). Overexpression of *dpod1* causes dose-dependent cytoskeleton remodeling (Rothenberg et al., 2003). These functions seemed to be conserved in mammals. Conditional *Coro7/crn7* knockout mice show alteration in F-actin content and orientation, changes in cell polarity and migration during wound healing, and in addition loss of *crn7* disrupts Golgi morphology (Bhattacharya et al., 2016). Mammalian *Coro7*, upon phosphorylation by Src kinase, becomes membrane-bound and is found to be localized in trans-Golgi, suggesting additional cellular localization and functions of *Coro7* besides cytoskeleton remodeling (Rybakin et al., 2004, 2008). To regulate cytoskeleton remodeling, the actin-binding *Coro7* interacts with Rho GTPase Cdc42 and N-WASP (Bhattacharya et al., 2016). In human cell culture, *Coro7* binds the core kinase complex and activates the Hippo pathway upon cell adhesion, serum deprivation, and injury to the cytoskeleton, suggesting that *Coro7* responds to environmental changes and stress conditions (Park et al., 2021).

Interestingly, the *Coro7/crn7* mRNA is downregulated in the mouse hypothalamus upon food deprivation (Eriksson et al., 2015). Conversely, stimulating appetite increases the *Coro7* positive neurons in the mouse locus coeruleus, and the *Coro7/dpod1* mRNA is upregulated in *Drosophila* upon overfeeding (Eriksson et al., 2015). Strikingly, obese children show lower methylation of a polymorphism close to *Coro7* compared to normal-weight children (Eriksson et al., 2015). This suggests that *Coro7* levels might be regulated by nutritional cues. We found that *Coro7*, positively and negatively correlated with lifespan duration, however, a dataset of *Coro7* in the brain or more specifically of the locus coeruleus might reveal a clearer picture. It might be that the longer-lived mice either consume less or have altered metabolism leading to longevity and *Coro7* levels might be just a readout for this. Beyond being a potential read-out for longevity, a previous study found that *Coro7/pod-1* knockdown extended *C. elegans’* lifespan by 45% in a *FOXO/daf-16-*dependent-manner (Kim and Sun, 2007). Whether the beneficial effects for healthy aging of Coro7 are mediated through endocytosis, altering Golgi functions, or cytoskeleton remodeling are important future experiments to be addressed.

Cytoskeleton remodeling has recently been shown to be essential for stress resistance and longevity in yeast, *C. elegans*, and *Drosophila* (Gourlay et al., 2004; Kumsta et al., 2013; Baird et al., 2014; Kaushik et al., 2015). We found strong longevity-associated enrichment for cytoskeleton remodeling in liver proteomics and bone transcriptomics (Figure 3). The cytoskeleton is anchored to cell surface receptors, such as integrins, which bind collagens and other extracellular matrix (ECM) proteins. Cytoskeleton remodeling often leads to ECM remodeling, a process important for mechanotransduction and healthy aging (Ewald et al., 2015; Ewald, 2019; Statzer and Ewald, 2020). We also observed a strong longevity-associated enrichment for ECM components in the kidney transcriptomics (Figure 3), highlighting mechanotransduction as a process potentially involved in longevity. Consistent with previous BXD mice analysis, we found mitochondrial components and metabolism (OXPHOS) to correlate with aging (Houtkooper et al., 2013; Williams et al., 2016, 2018, 2021). Interestingly, we found the bZIP transcription factor *Atf5* to correlate with longevity in bones (Figure 2). The *Atf5* is the *C. elegans atfs-1* ortholog essential for the mitochondrial unfolded protein response (Fiorese et al., 2016), which is vital for healthy aging (Houtkooper et al., 2013; Wu et al., 2018). A more surprising finding was the enrichment for neuronally expressed genes, such as amyloid precursor protein *APP* (Supplementary Figure 4), a gene causally implicated in Alzheimer’s disease (Selkoe, 1998). The *C. elegans* ortholog of *APP*, when expressed from the hypodermis, increases *C. elegans* lifespan (Ewald et al., 2016). This raises the possibility that neuronal-expressed genes in other tissues might play a functional role in promoting healthy aging. Thus, our analysis revealed several previously established and less well-studied longevity-promoting processes.

The unique sex- and tissue-specific longevity-correlating gene signatures are two tantalizing observations from our study that should be addressed in future studies. Our study’s possible limitations are that there might be not enough comparative data, variability of different sequencing techniques from different labs, or that multiple tissues were not from the same individual. Another factor could be the heterogeneity and different genetic background of the individual BXD mice. It could also be that different tissues express different splice variants that we can not distinguish with our analysis. Lastly, the differences might be due to the observation that each tissue ages differently, and heterogeneity in random molecular damage accumulates at different rates in different tissues, leading to tissue- and sex-specific adaptations However, we found in independent wild-type mouse datasets that at least for the most common longevity-correlating genes (*Coro7* and *Set*) also showed sex- and tissue-specific gene signatures. Furthermore, sex differences for longevity are recently more reported (Mitchell et al., 2016). For instance, a recent study found sex-specific single nucleotide polymorphism for human longevity genes, including the most common identified longevity loci *TOMM40* and *APOE* (Liu et al., 2021). Thus, future studies should attempt to parse the data in a sex-specific manner to more accurately determine these gender-specific signatures.

In summary, we identified novel longevity-promoting genes in omics datasets of the well-characterized BXD mouse family. We demonstrate that a systems-level biological framework identifies several known and established longevity genes and pathways, and implicates roles for *Set* and *Coro7* in longevity. Finally, we provide evidence that genes that correlate with lifespan do so in a tissue- and sex-specific manner. Dissecting these tissue- and sex-specific gene expression correlations may significantly impact our understanding of the different regulation of longevity networks that promote healthy aging.

## Data Availability Statement

The original contributions presented in the study are included in the article/Supplementary Material, further inquiries can be directed to the corresponding author.

## Author Contributions

CE and DV designed the study and wrote the manuscript in consultation with AD and CS. DV performed the computational analysis. AD performed literature searches. All authors participated in analyzing and interpreting the data.

## Conflict of Interest

The authors declare that the research was conducted in the absence of any commercial or financial relationships that could be construed as a potential conflict of interest.

## Footnotes

1 http://gn1.genenetwork.org/webqtl/main.py?FormID=sharinginfo&GN_AccessionId=239&InfoPageName=MA_M2F_0706_R

2 http://gn1.genenetwork.org/webqtl/main.py?FormID=sharinginfo&GN_AccessionId=207&InfoPageName=Eye_M2_0908_R

3 http://gn2.genenetwork.org/
